# Sleep Disturbances and Health-Related Quality of Life in Adults with Steady-State Bronchiectasis

**DOI:** 10.1371/journal.pone.0102970

**Published:** 2014-07-18

**Authors:** Yonghua Gao, Weijie Guan, Gang Xu, Zhiya Lin, Yan Tang, Zhimin Lin, Huimin Li, Yang Gao, Qun Luo, Nanshan Zhong, Rongchang Chen

**Affiliations:** State Key Laboratory of Respiratory Diseases, National Clinical Research Center for Respiratory Disease, Guangzhou Institute of Respiratory Diseases, The First Affiliated Hospital of Guangzhou Medical University, Guangzhou, Guangdong, China; University of Rochester, United States of America

## Abstract

**Background:**

Sleep disturbances are common in patients with chronic lung diseases, but little is known about the prevalence in patients with bronchiectasis. A cross sectional study was conducted to investigate the prevalence and determinants associated with sleep disturbances, and the correlation between sleep disturbances and quality of life (QoL) in adults with steady-state bronchiectasis.

**Methods:**

One hundred and forty-four bronchiectasis patients and eighty healthy subjects were enrolled. Sleep disturbances, daytime sleepiness, and QoL were measured by utilizing the Pittsburgh Sleep Quality Index (PSQI), Epworth Sleepiness Scale (ESS) and St. George Respiratory Questionnaire (SGRQ), respectively. Demographic, clinical indices, radiology, spirometry, bacteriology, anxiety and depression were also assessed.

**Results:**

Adults with steady-state bronchiectasis had a higher prevalence of sleep disturbances (PSQI>5) (57% vs. 29%, P<0.001), but not daytime sleepiness (ESS≥10) (32% vs. 30%, P = 0.76), compared with healthy subjects. In the multivariate model, determinants associated with sleep disturbances in bronchiectasis patients included depression (OR, 10.09; 95% CI, 3.46–29.37; *P*<0.001), nocturnal cough (OR, 1.89; 95% CI, 1.13–3.18; *P* = 0.016), aging (OR, 1.04; 95% CI, 1.01–1.07; *P* = 0.009) and increased 24-hour sputum volume (OR, 2.01; 95% CI, 1.22–3.33; *P* = 0.006). Patients with sleep disturbances had more significantly impaired QoL affecting all domains than those without. Only 6.2% of patients reported using a sleep medication at least weekly.

**Conclusions:**

In adults with steady-state bronchiectasis, sleep disturbances are more common than in healthy subjects and are related to poorer QoL. Determinants associated with sleep disturbances include depression, aging, nighttime cough and increased sputum volume. Assessment and intervention of sleep disturbances are warranted and may improve QoL.

## Introduction

Bronchiectasis, characterized by irreversible bronchial dilatation in patients suffering from productive cough, purulent sputum and recurrent infective exacerbations, has attracted insufficient attention to date, presumably due to the perception that significant bronchiectasis is uncommon and there is no specific therapy when identified [1]–[4]. Considering the paucity of evidence-based data, treatment of bronchiectasis remains largely empirical, or extrapolated from miscellaneous respiratory diseases, i. e. cystic fibrosis (CF) and chronic obstructive pulmonary disease (COPD) [1]. This underscored the importance for further research to investigate appropriate management for this neglected disease and improve patient’s quality of life (QoL) [5].

Sleep disturbances are common among chronic lung diseases (i.e. COPD, CF and idiopathic pulmonary fibrosis) and contribute substantially to fatigue, depression and impaired QoL [6]–[8]. To date, the literature on this topic in bronchiectasis has been limited. A recent study [9] reported that children with bronchiectasis had a high prevalence of sleep disturbances associated with nocturnal respiratory symptoms and disease severity. However, apart from focusing on children, the sample size in their study was insufficient to warrant a conclusive finding, and the potential impact of psychological status on sleep disturbances was also unclear. QoL was reportedly impaired in patients with bronchiectasis [7], at least partially due to sleep disturbances, as evidenced in other chronic diseases [8], [10]–[12]. Whether QoL in those with bronchiectasis and concomitant sleep disturbances is worse than that in patients with bronchiectasis but no sleep disturbances remains unknown.

In this study, we sought to: (1) determine the prevalence of sleep disturbances in adults with steady-state bronchiectasis and compare this rate with healthy subjects; (2) delineate the determinants associated with sleep disturbances; (3) elucidate the effect of sleep disturbances on QoL assessed by the St George' Respiratory Questionnaire (SGRQ).

## Materials and Methods

### Study design and participants

One hundred and forty-four patients with steady-state bronchiectasis were recruited from the out-patient clinic of First Affiliated Hospital of Guangzhou Medical University, between September 2012 and April 2013. Bronchiectasis was diagnosed by the presence of a compatible history combined with bronchial dilatation on high-resolution computed tomography (HRCT) [1]. Steady-state bronchiectasis was defined as the absence of clinical deterioration (i.e. increased cough, sputum volume or dyspnea) beyond normal daily variations within the last 4 weeks prior to evaluation. Exclusion criteria were patients: (1) aged 18 years or less; (2) had acute exacerbation within 1 month; (3) uncontrolled asthma; (4) traction bronchiectasis due to severe emphysema or advanced fibrosis; (5) poor understanding of the questionnaire; (6) other medical conditions leading to sleep disturbances (i.e. pain or previously diagnosed sleep disorders).

80 healthy subjects who were individually matched with the first 80 consecutive bronchiectasis patients for age and sex were recruited during the same period from the Health Check-up Center of First Affiliated Hospital of Guangzhou Medical University. Healthy subjects were evaluated by careful medical history inquiry with special attention to the previous history of respiratory diseases or current respiratory symptoms. Spirometry and thoracic HRCT were not mandatory. Exclusion criteria included malignant tumors, chronic systemic diseases and conditions that could affect sleep quality (i.e. working in night shifts in the last 1 month).

The study was approved by the Ethics Committee of the First Affiliated Hospital of Guangzhou Medical University. All subjects provided written informed consent.

### Main outcomes

Sleep disturbances and daytime sleepiness were the main outcomes of assessment. All patients completed the Chinese version of the Pittsburgh Sleep Quality Index (PSQI), a validated self-report measure that assessed 7 sleep domains (subjective sleep quality, sleep latency, sleep duration, habitual sleep efficiency, sleep disturbances, use of sleep medications and daytime dysfunction ) over the preceding month, with an ideal diagnostic power for a global score of 6 or more in differentiating poor from good sleepers [13], [14].

A validated Chinese version of Epworth Sleepiness Scale (ESS) was used to measure excessive daytime sleepiness in 8 conditions using a 0–3 scale, with a total score ranging from 0 to 24 [15]. Daytime sleepiness was defined as a total score of 10 or greater [16].

### Additional data Collected

A comprehensive history including age, sex, body-mass index (BMI), smoking history, education level, employment, marital status, disease duration, previous history and current treatments was collected in the first interviewer-conducted survey. The etiology of bronchiectasis was determined after meticulous testing recommended by British Thoracic Society guidelines^1^ and thorough group discussion (Y.G., W.G. and G.X.). Rhinosinusitis was defined by the criteria recommended by EP^3^OS guidelines [17]. The number of infective exacerbations in previous 12 months was extracted by meticulous history taking and review of the clinical records. Patients were instructed to collect sputum over 24 hours in a sterile plastic pot (50 ml), and then semi-quantitative evaluation of 24-hour sputum volume (<10 ml, 10–30 ml or >30 ml) was determined by three consecutive daily records during hospital visits by a designated technician (H.L.). Cough was assessed by a cough symptom score consisting of daytime and night-time cough symptoms scale (0–5 points) based on the severity [18]. The total cough symptom scores were derived by summation of the day-time and night-time scores, with a maximum possible score of 10. Baseline dyspnea was assessed by using modified Medical Research Council scale (MRC), a standardized 4-point scale from Grade 1 (breathless with strenuous exercise) to Grade 5 (too breathless to leave the house or when dressing) [19]. The severity of bronchiectasis was assessed by scoring HRCT scans by a modified Reiff score, with the maximal possible score of 18 for 6 lung lobes [20].

Anxiety and Depression Assessment: a validated Chinese-Cantonese version of Hospital Anxiety and Depression Scale (HADS) was applied to measure anxiety and depression status [21]. HADS was consisted of 7 items for depression (HAD-D) and 7 for anxiety (HAD-A), each of which was scored on a scale of 0–3. For the scores for both subscales, 0–7 denoted normal, 8–10 possible and 11–21 probable [22].

QoL was assessed based on SGRQ which has been previously validated for use in bronchiectasis patients [23], [24]. The SGRQ contained 50 items divided into three domains: symptoms, activities, and impacts. The total and individual domain scores ranged from 0 to 100, with a higher score representing worse QoL. The minimal clinically important difference (MCID) for SGRQ was 4 units [17],[18].

Lung Function: Please refer to File S1.

Sputum bacteriology: Please refer to File S1.

### Statistical analyses

Descriptive statistics were tabulated as mean (standard deviation, SD) or median (interquartile range, IQR) or counts (proportion), as appropriate. Two-group comparisons were performed using unpaired t-tests, Mann-Whitney U tests or chi-square tests, when appropriate. Univariate and multivariate associations of determinants (listed above) with poor sleep quality (PSQI>5) were identified by logistic regression model. Odds ratios (OR) for the prevalence of sleep disturbances were estimated with 95% confidence intervals (CIs). Determinants with a P value of 0.10 or less in univariate models were initially included in the multivariate model and were then discarded using backward selection. Spearman’s rank correlation was used to assess the correlation of PSQI total score and HADS-depression score. All comparisons were two-sided, with P values <0.05 being considered statistically significant. Statistical analyses were performed using SPSS 17.0 (Chicago, IL, USA) and Graphpad Prism Version 5.0 (Graphpad Software, San Diego, CA, USA).

## Results

### Subject characteristics

We evaluated 144 bronchiectasis patients aged 46.2 (13.6) years, of whom 62% were females (Table 1). The mean BMI of bronchiectasis patients was 20.3 (3.1) kg/m^2^, which was lower than that of healthy subjects [23.4 (2.7) kg/m^2^]. In terms of HADS, an increased number of bronchiectasis patients were characterized by elevated scores for anxiety [67 (46.5%) vs. 5 (6.3%), P<0.001] and depression [43 (29.9%) vs. 8 (10.0%), P = 0.003] compared with healthy subjects. Patients with bronchiectasis had a FEV_1_ of 67.4% (22.6%) predicted. And 102 patients (70.8%) had idiopathic/post-infective bronchiectasis in our cohort.

**Table 1:** Clinical characteristics of the study population.

	No. (%)^a^		
Characteristic	Bronchiectasis (n = 144)	Healthy subjects (n = 80)	P value*
**Age, mean (SD), y**	46.2 (13.6)	47.1 (12.3)	0.62
**Female sex**	89 (61.8)	47 (58.8)	0.65
**Body-mass index, mean (SD), kg/m^2^**	20.3 (3.1)	23.4 (2.7)	<0.001
**Smoking status**			<0.001
Current	3 (2.1)	22 (27.5)	
Former	20 (13.9)	2 (2.5)	
Never	121 (84.0)	56 (70.0)	
**Marital status**			0.12
Never married	15 (10.4)	14 (17.5)	
Married/cohabiting	119 (82.6)	65 (81.2)	
Divorced/separated	4 (2.8)	1 (1.3)	
Widowed	6 (4.2)	0 (0.0)	
**Education**			
Highest completed level	66 (45.9)	38 (47.1)	
Less than high school	42 (29.2)	24 (30.2)	
High school graduate or equivalent	36 (25.0)	18 (22.7)	
College graduate or above			
**Employment status**			0.001
Working full time or part time	70 (48.6)	51 (63.8)	
Not working due to health	15 (10.4)	0 (0.0)	
Student	2 (1.4)	6 (7.5)	
Housewife	9 (6.3)	4 (5.0)	
Seeking work/not work for other reasons	48 (33.3)	19 (23.8)	
**Anxiety and Depression**			
HADS-Anxiety score≥8	67 (46.5)	5 (6.3)	<0.001
HADS-Depression score≥8	43 (29.9)	8 (10.0)	0.003
**Pulmonary function, mean (SD)**			
FEV_1_ pred%	67.4 (22.6)	…	…
FVC pred%	76.5 (19.4)	…	…
**Rhinosinusitis**	40 (27.8)	…	…
**Cause of bronchiectasis**			
Idiopathic/post infective	102 (70.8)	…	…
Humoral immune deficiency	10 (6.9)	…	…
COPD or asthma	9 (6.3)	…	…
Kartagener syndrome	3 (2.1)	…	…
Previous ABPA	2 (1.4)	…	…
Rheumatoid arthritis	2 (1.4)	…	…
Others	16 (11.1)	…	…

Abbreviations: ABPA = Allergic bronchopulmonary aspergillosis; COPD = Chronic obstructive pulmonary disease; FEV1 =  Forced expiratory volume in one second; FVC = Forced vital capacity; HADS = Hospital anxiety and depression scale; pred = Predicted values; SD = Standard deviation. ^a^ Unless otherwise specified, values represent no. (%) of participants.

*Unpaired t test or chi-square test was used to compare variables in bronchiectasis patients vs. controls as appropriate.

### Sleep disturbances in bronchiectasis patients compared with healthy subjects

Data of ESS and PSQI, including subjective sleep quality, sleep latency, sleep duration, sleep efficiency, sleep disturbance, use of sleep medications and daytime dysfunction, are summarized in Table 2. Except for sleep duration and use of sleep medication, the remaining domains of PSQI showed pronounced between-group differences. 82 bronchiectasis patients (56.9%; 95% CI, 48.8%–65.0%) had sleep disturbances based on PSQI (>5), which was significantly higher than those in healthy subjects (28.8%; 95% CI, 21.4%–36.2%; P<0.001). Regarding the ESS, 46 bronchiectasis patients (31.9%; 95% CI, 24.3%–39.5%) evidenced daytime sleepiness (≥10), of which the difference was insignificant compared with that of healthy subjects (30.0%; 95% CI, 22.5%–37.5%; P = 0.76). Finally, patients with bronchiectasis rarely received medications for sleep despite a considerably high incidence of sleep disturbances.

**Table 2:** Pittsburgh Sleep Quality Index (PSQI) and Epworth Sleepiness Scale (ESS) scores in bronchiectasis patients and health subjects.

	Patients with bronchiectasis*	Healthy Controls*	
Sleep characteristic	N = 144	N = 80	P values†
**Sleep duration**			
Self-reported sleep duration (h)	7 (6.0–7.5)	7 (5.6–8.0)	0.85
**Sleep quality**			
PSQI total score	6 (4.0–11.0)	4 (3.0–6.0)	<0.001
PSQI>5, n (%)	82 (56.9)	23 (28.8)	<0.001
Component score			
1, Subjective sleep quality	1 (1.0–2.0)	1 (1.0–1.0)	0.003
2, Sleep latency	1 (0.0–2.0)	1 (0.0–1.0)	<0.001
3, Sleep duration	1 (0.0–2.0)	1 (0.0–1.0)	0.14
4, Sleep efficiency	0 (0.0–2.0)	0 (0.0–1.0)	<0.001
5, Sleep disturbances	1 (1.0–2.0)	1 (0.0–1.0)	<0.001
6, Use of sleep medication	0 (0.0–0.0)	0 (0.0–0.0)	0.40
7, Daytime dysfunction	1 (1.0–2.0)	1 (1.0–2.0)	0.05
**Daytime sleepiness**			<0.001
ESS total score	7 (4.0–11.0)	7 (5.0–10.0)	0.93
Sleepy, ESS≥10, n (%)	46 (31.9)	24 (30.0)	0.76
Very sleepy, ESS≥18, n (%)	6 (4.2)	0 (0.0)	0.09

Abbreviations: ESS = Epworth Sleepiness Scale; h = Hours; PSQI = Pittsburgh Sleep Quality Index.

*Data are presented as median (IQR) or no. (%) as appropriate.

† Mann-Whitney U test was used to compare continuous variables, and chi-square test was used to compare category variables in patients with bronchiectasis vs. healthy controls.

Of patients who responded to “Do you feel that your sleep has been disturbed by cough or sputum production?” during history inquiry, 61 reported sleep disturbance by cough or sputum production, of whom 47 were graded as poor and 14 good sleepers by the global PSQI.

### Determinants of sleep disturbances in bronchiectasis patients

Among patients with steady-state bronchiectasis, univariate analyses showed that all possible variables associated with sleep disturbances (PSQI>5) included aging (OR, 1.03 per year; 95% CI, 1.01–1.06;P = 0.012), reduced BMI (OR, 0.88; 95% CI, 0.78–0.98; p = 0.02), depression (OR, 7.67; 95% CI, 2.98–19.80; P<0.001), anxiety (OR, 2.87; 95% CI, 1.41–5.88; P = 0.003), 24-hour sputum volume>30 ml (OR, 2.31; 95% CI, 1.01–5.29; P = 0.05), increased daytime cough symptom score (OR, 1.77; 95% CI, 1.22–2.57; P = 0.003) and nighttime cough symptom score (OR, 2.10; 95% CI, 1.35–3.27; P = 0.001), airway colonization with Pseudomonas *aeruginosa* (OR, 2.44; 95% CI, 1.06–5.65; P = 0.03), and severe dyspnea (OR, 4.68; 95% CI, 1.51–14.50; P = 0.004) (Table 3). Patients with the education level of college graduate or above (OR, 0.41; 95% CI, 0.18–0.94; P = 0.03) had a lower prevalence of sleep disturbances compared with those who had education level of high school or below. Furthermore, seeking jobs or not working for other reasons (OR, 2.47; 95% CI, 1.14–5.33; P = 0.02) was linked to a higher prevalence of sleep disturbances.

**Table 3:** Determinants associated with sleep disturbances in bronchiectasis patients.

	Poor sleepers, No. (%)^a^		Univariate Models	
Determinants	Yes (n = 82)	No (n = 62)	OR (95% CI)	P value*
**Age, mean (SD), yr**	48.7 (12.8)	42.9 (14.0)	1.03 (1.01–1.06)	0.012
**Sex**				
Female	50 (61.0)	39 (62.9)	0.92 (0.47–1.82)	0.81
Male	32 (39.0)	23 (37.1)	1.00	…
**BMI, kg/m^2^**				
**<18.5**	32 (39.0)	14 (22.6)	2.17 (1.01–4.69)	0.05
18.5–23.9	40 (48.8)	38 (61.3)	1.00	…
** ≥**24	10 (12.2)	10 (16.1)	0.95 (0.36–3.54)	0.51
**Smoking status**				
Current	2 (2.4)	1 (1.6)	1.56 (0.14–17.66)	1.00
Former	12 (14.6)	8 (12.9)	1.17 (0.45–3.07)	0.75
Never	68 (82.9)	53 (85.5)	1.00	…
**Marital status**				
Never married	8 (9.8)	7 (11.3)	0.85 (0.29–2.52)	0.78
Married/cohabiting	68 (82.9)	51 (82.3)	1.00	…
Divorced/separated	2 (2.4)	2 (3.2)	0.75 (0.10–5.51)	1.00
Widowed	4 (4.9)	2 (3.2)	1.50 (0.26–8.51)	1.00
**Education**				
Highest completed level				
Less than high school	42 (51.2)	24 (38.7)	1.00	…
High school graduate or equivalent	25 (30.5)	17 (27.4)	0.84 (0.38–1.86)	0.67
College graduate or above	15 (18.3)	21 (33.9)	0.41 (0.18–0.94)	0.03
**Employment status**				
Working full time or part time	33(40.2)	37(59.7)	1.00	…
Not working due to health	10(12.2)	8(8.1)	1.40(0.50–3.97)	0.52
Student	0(0)	2(3.2)	…	…
Housewife	6(7.3)	3(4.8)	2.24(0.52–9.69)	0.27
Seeking work/not work for other reasons	33(40.2)	15(24.2)	2.47(1.14–5.33)	0.02
**Cough symptom score, median (IQR)**				
Daytime score	2 (2.0–3.0)	2 (1.0–2.0)	1.77 (1.22–2.57)	0.003
Nighttime score	1 (1.0–2.0)	1 (0–1.0)	2.11 (1.35–3.27)	0.001
**Depression (HADS-D≥8)**				
** Yes**	37 (45.1)	6 (9.7)	7.67 (2.98–19.80)	<0.001
No	45 (54.9)	56 (90.3)	1.00	…
**Anxiety (HADS-A≥8)**				
** Yes**	41 (50.0)	16 (25.8)	2.87 (1.41–5.88)	0.003
No	41 (50.0)	46 (74.2)	1.00	…
**Onset of productive cough in childhood**				
Yes	15 (18.3)	12 (19.4)	0.93 (0.40–2.17)	0.87
No	67 (81.7)	50 (80.6)	1.00	…
**FEV_1_<50% pred**				
Yes	17 (20.7)	14 (22.6)	0.90 (0.40–2.00)	0.79
No	65 (79.3)	48 (77.4)	1.00	…
**HRCT score**				
<6	35 (42.7)	22 (35.5)	1.00	…
6–12	37 (45.1)	33 (53.2)	0.71 (0.35–1.43)	0.33
** ≥**12	10 (12.2)	7 (11.3)	0.90 (0.30–2.71)	0.85
**Daily sputum**				
<10 ml	29 (35.4)	30 (48.4)	1.00	…
10–30 ml	24 (29.3)	19 (30.6)	1.31 (0.59–2.88)	0.51
**>30** **ml**	29 (35.4)	13 (21.0)	2.31 (1.01–5.29)	0.05
**PA colonization**				
** Yes**	25 (30.5)	10 (16.1)	2.44 (1.06–5.65)	0.03
No	48 (58.5)	47 (75.8)	1.00	…
No information	9 (11.0)	5 (8.1)	1.76 (0.55–5.65)	0.34
**MRC dyspnea scale**				
0–1	62 (75.6)	58 (93.5)	1.00	…
** ≥2**	20 (24.4)	4 (6.5)	4.68 (1.51–14.50)	0.004
**Exacerbations in previous year**				
0–2	62 (73.2)	48 (74.2)	1.00	…
** ≥**3	20 (22.0)	14 (19.4)	1.11 (0.51–2.41)	0.80
**Rhinosinusitis**				
Yes	24 (29.3)	16 (25.8)	1.19 (0.57–2.50)	0.65
No	58 (70.7)	46 (74.2)	1.00	…

Abbreviations: BMI = Body mass index; CI = Confidence interval; FEV1 =  Forced expiratory volume in one second; HADS-A = Hospital anxiety and depression scale-anxiety; HADS-D = Hospital anxiety and depression scale-depression; HRCT = High-resolution computed tomography; IQR = Interquartile range; OR = Odds ratios; PA = Pseudomonas aeruginosa; pred = Predictive value; MRC = Medical Research Council Scale; SD = Standardized deviation; yr = Year.

a Data were presented as no. (%), unless otherwise specified.

*Univariate Logistic analysis was conducted to explore the determinants of sleep disturbances, and results were reported as odds ratio (OR) and 95% confidence interval (CI).

In the multivariate model (Table 4), determinants associated with sleep disturbances, based on the PSQI, included depression (OR, 10.09; 95% CI, 3.46–29.37; P<0.001), increased 24-hour sputum volume (>30 ml) (OR, 2.01; 95% CI, 1.22–3.33; P = 0.006), increased nighttime cough symptom (OR, 1.89; 95% CI, 1.13–3.18; P = 0.016) and aging (OR, 1.04; 95% CI, 1.01–1.07; P = 0.009).

**Table 4:** Multivariate analysis of determinants associated with sleep disturbances (PSQI>5) in bronchiectasis patients.

	Sleep disturbances (PSQI>5)		
Determinants	OR	95% CI	P value†
**Age**	1.04	1.01–1.07	0.009
**Increased 24-hour sputum volume (>30** **ml)**	2.01	1.22–3.33	0.006
**Night cough symptom score**	1.89	1.13–3.18	0.016
**Depression (HADS-D≥8)**	10.09	3.46–29.37	<0.001

† In multivariate analysis, determinants with a P value ≤0.1 in preceding univariate analyses, including age, BMI, education, employment status, depression score, anxiety score, 24-hour sputum volume, daytime cough score, nighttime cough score, PA colonization, and MRC dyspnea score, were initially included, and were then eliminated using backward selection.

**Figure 1:**
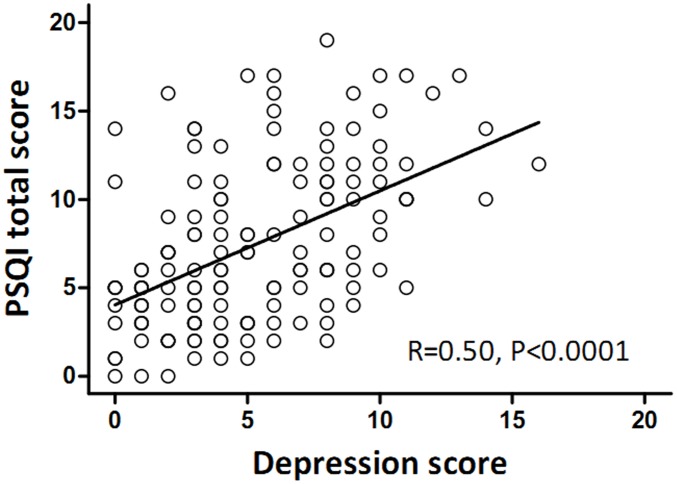
Correlation between depression and PSQI total score in bronchiectasis patients.

Regarding the association of sleep disturbance and depression evaluated by HADS-D, sleep disturbance was present in 44.6% of patients without depression, 84.4% with possible depression and 90.9% with probable depression (P<0.001). Moreover, higher HADS-D scores were significantly associated with increased PSQI scores (Spearman correlation coefficient, 0.50; P<0.001, Figure 1). This group of patients with bronchiectasis rarely took antidepressant medications despite the high incidence of depression.

### Impact of sleep disturbances on QoL in bronchiectasis patients

The SGRQ, including 3 individual domains (symptom, activity and impact), was employed to measure the QoL in bronchiectasis patients. There was a significant association between sleep quality and QoL both in individual domain scores and total scores; with those who had sleep disturbances demonstrating more significantly impaired QoL. The actual domain scores and total scores in poor sleepers (PSQI>5) were pronouncedly higher than those in good sleepers (PSQI≤5): symptom domain [37.5 (25.6–52.8) vs. 27.0 (19.0–51.0), P = 0.003]; activity domain [34.6 (21.4) vs. 23.3 (19.8), P = 0.002]; impact domain [40.7 (19.6) vs. 26.2 (17.2), P<0.001]; total scores [39.3 (17.4) vs. 26.6 (15.8), P<0.001] (Table 5).

**Table 5:** Comparisons of SGRQ domain scores and total scores between poor (PSQI>5) and good sleepers(PSQI≤5) in bronchiectasis patients.

SGRQ scores	Poor sleepers (n = 82)	Good sleepers (n = 62)	P values
**Symptom**	37.5 (25.6–52.8)	27.0 (19.0–51.0)	0.003*
**Activity**	34.6 (21.4)	23.3 (19.8)	0.002†
**Impact**	40.7 (19.6)	26.2 (17.2)	<0.001†
**Total**	39.3 (17.4)	26.6 (15.8)	<0.001†

Data were presented as mean (SD) or median (IQR), as appropriate.

*Mann Whitney U test was used to compare the SGRQ symptom scores between good sleepers and poor sleepers.

† Unpaired t test was used to compare the SGRQ activity, impact, and total scores between good sleepers and poor sleepers.

## Discussion

This is, to our knowledge, the first study to investigate the prevalence of sleep disturbances and daytime sleepiness, and their relationship with QoL in adults with bronchiectasis by utilizing standardized questionnaires [9]. Although daytime sleepiness did not differ considerably from healthy subjects, the prevalence of sleep disturbances was an approximately 2-fold higher in bronchiectasis patients. Aging, nocturnal cough, increased 24-hour sputum volume and presence of depression were associated with a higher prevalence of sleep disturbances. Furthermore, sleep disturbances was, in turn, associated with reduced QoL as assessed by SGRQ.

To date, there has been only one study evaluating sleep quality in bronchiectasis with an insufficient sample size in children, which reported that children with bronchiectasis had a higher prevalence of sleep disturbances (up to 37%) [9]. This necessitated further study in adult patients. Our study consequently focused on adults with bronchiectasis in order to elucidate the prevalence of sleep disturbances, and delineate the potential relationships between demographic, disease-related and psychological variables and sleep quality by using multivariable regression models.

The major determinant associated with sleep disturbances among adults with bronchiectasis explicitly pointed to the presence of depression. This association remained strong, even after adjustment of demographic and disease-specific variables. This confirmed the previous findings in general populations and other chronic diseases [25]–[28], demonstrating that psychological co-morbidity constituted the major determinant in sleep disturbances among adults with bronchiectasis. Considering the multi-faceted association between sleep quality and depression, the cross-sectional nature of our study inevitably made it difficult to determine the causal association between sleep and depression. This has led us to postulate that treatment of depression may result in ameliorated conditions of sleep disorders, and the vice versa [29].

Increased nocturnal cough and 24-hour sputum volume were also pertinent to sleep disturbances. In daily practice, bronchiectasis patients seldom complained of cough without sputum production, suggesting that cough frequently coincided with sputum production [30]. Previous studies documented that cough or sputum production was associated with an increased risk of sleep disturbances and daytime sleepiness in a general population [31]. Meanwhile, Chan et al [32] reported that chronic cough was a crucial symptom linked to sleep-disordered breathing. In addition, nocturnal cough may delay patients’ progression to a deeper stage of sleep and rapid eye movement (REM) sleep, a condition frequently happened in patients with CF [33]. Unfortunately, the fact that polysomnography (PSG) and 24-hour ambulatory cough monitoring were not performed has rendered it difficult for the present study to fully explore the exact causes of aforementioned associations.

Regarding disease-related variables, our findings showed that there were no significant association between sleep quality and disease severity, assessed by spirometry, dyspnea scale, exacerbation frequency and Pseudomonas *aeruginosa* colonization in the final multivariate model. This indicated that sleep disturbances in bronchiectasis patients might have resulted from distinct mechanisms not related to spirometry or disease severity. It could also be the interpretation that miscellaneous factors (i.e. aging, cough, sputum production and psychiatric co-morbidities) affect overall sleep quality. In addition, we did not find a correlation between sleep quality and HRCT scores. A previous study conducted in children with bronchiectasis documented a positive correlation between HRCT scores and sleep quality assessed by using PSQI [9]. The divergence of these findings may be due to, at least in part, the differences in the subjects investigated.

As reported in cross-sectional studies of patients with miscellaneous chronic respiratory diseases [6], [8], [10], [11], our study found a substantially reduced disease-specific QoL in poor sleepers compared with good sleepers in bronchiectasis patients. The magnitude of these differences was clinically significant because they far exceeded the 4 units’ threshold for MCID in the SGRQ [17], [18]. One potential explanation for these findings might be a higher risk of presence of anxiety and depression symptoms in patients with poor sleep quality, which has been associated with poorer health-related QoL experienced by these individuals [34], [35]. In addition, increased cough symptom and 24-hour sputum volume in patients with poor sleep quality also worsens the QoL. Despite the high prevalence of sleep disturbances in bronchiectasis, only 6.2% were using a medication for sleep once weekly or more. Considering the current limited treatment for improving QoL in bronchiectasis, our data suggested that interventions to improve sleep or associated risk factors would act positively to the lifestyle in bronchiectasis patients with concomitant sleep disturbances.

Several potential limitations must be considered. First, we assessed the sleep disturbances based on questionnaire survey rather than PSG, therefore the impact of other sleep disorders, i.e. obstructive sleep apnea (OSA), might have been underestimated. However, it was unlikely that OSA appeared more frequently in our cohort since BMI was lower than that of healthy subjects, and the cost of PSG has limited the utility as a screening tool for individual participant. Second, this was a cross-sectional, rather than prospective, study, which was unable to elucidate the casual relationships between sleep disturbance and determinants or reduced QoL in bronchiectasis, nor for the assessment of whether sleep disturbances were transient or chronic. Third, though nocturnal cough was associated with sleep disturbances, the exact association could not be established due to the lack of objective measures, i.e. 24-hour ambulatory cough monitoring and PSG. Finally, this study was conducted at a single tertiary hospital, which might have limited the generalisability of our results. Therefore, a large multicenter community-based study may further confirm the magnitude of sleep disturbances and its impact on QoL in bronchiectasis.

In summary, sleep disturbances are common in adults with steady-state bronchiectasis compared with healthy subjects and are independently associated with depression, increased nighttime cough, increased 24-hour sputum volume and aging. Unsurprisingly, sleep disturbances are significantly associated with reduced QoL. Therefore, interventions targeting at promoting sleep may improve the QoL in these patients who currently have very limited therapeutic options.

## Supporting Information

File S1 **Methods of lung function and sputum bacteriology.**
(DOCX)
