# Supplementary material for: Sleep Disturbances and Health-Related Quality of Life in Adults with Steady-State Bronchiectasis
Source: PLoS One. 2014 Jul 18;9(7):e102970. doi: 10.1371/journal.pone.0102970 (PMC4103887; doi:10.1371/journal.pone.0102970)
Supplement: File S1 — Methods of lung function and sputum bacteriology. (DOCX) [file pone.0102970.s001.docx]

**Sleep disturbances and health-related quality of life in adults with steady-state bronchiectasis**

Yonghua Gao^*^, Ph.D.; Weijie Guan^*^, Ph.D.; Gang Xu^*^, Ph.D.; Zhiya Lin, M.D.; Yan Tang, M.D.; Zhimin Lin, M.M.; Huimin Li, M.T.; Yang Gao, Ph.D.; Qun Luo, Ph.D.; Nanshan Zhong, M.D.; Rongchang Chen^**^, M.D.

State Key Laboratory of Respiratory Diseases, National Clinical Research Center for Respiratory Disease, Guangzhou Institute of Respiratory Diseases, The First Affiliated Hospital of Guangzhou Medical University, Guangzhou, Guangdong, China.

***These three authors contributed equally to this paper.**

****Correspondence should be addressed to Prof. Rongchang Chen**, State Key Laboratory of Respiratory Diseases, Guangzhou Institute of Respiratory Diseases, The First Affiliated Hospital of Guangzhou Medical University, 151 Yanjiang Road, Guangzhou, Guangdong, 510120, China. E-mail: [ChenRC@vip.163.com](mailto:ChenRC@vip.163.com). Telephone: +862083062882; Fax: +862083062718

**Methods:**

Lung Function*:* Spirometry was performed, in which forced expiratory volume in 1 second (FEV_1_) and forced vital capacity (FVC) were derived, by using spirometers (QUARK PFT, COSMED Co. Ltd, Italy). The quality control met the recommendations by American Thoracic Society/European Respiratory Society [1]. Data were expressed as absolutes and percentage predicted using reference equations recommended by Zheng et al [2]. At least 3 technically satisfactory measurements were recorded, with the variation between the best two maneuvers of <5% or 150ml in FVC. The maximal values of FVC and FEV_1_ were reported.

Sputum bacteriology*:* Sputum culture reports from medical records in the preceding year were meticulously extracted. Following chest physiotherapy, spontaneous sputum samples before 10:00am containing less than 10 squamous cells and more than 25 leukocytes under microscope (magnification: *100) were collected and sent for bacteriology assay within 2 hours at 3-month intervals. Determination of Pseudomonas aeruginosa (P. aeruginosa) colonization, defined by an identical isolation on two or more occasions, at least 3 months apart within a 1-year period, was assessed in conjunction with previously available results.

**REFERENCE**

1. Miller MR, Hankinson J, Brusasco V, Burgos F, Casaburi R et al (2005) Standardisation of spirometry. *Eur Respir J.* 26(2): 319-38.

2. Zheng J, Zhong N (2002) Normative values of pulmonary function testing in Chinese adults. *Chin Med J (Engl).* 115(1): 50-4.
